# 3,3,3-Trifluoro-2-hydr­oxy-2-(trifluoro­meth­yl)propionic acid

**DOI:** 10.1107/S1600536813003103

**Published:** 2013-02-02

**Authors:** Thomas Gerber, Richard Betz

**Affiliations:** a Nelson Mandela Metropolitan University, Summerstrand Campus, Department of Chemistry, University Way, Summerstrand, PO Box 77000, Port Elizabeth, 6031, South Africa

## Abstract

In the title perfluorinated hy­droxy­isobutyric acid derivative, C_4_H_2_F_6_O_3_, the mol­ecule shows approximately *C*_s_ symmetry. The carb­oxy group is nearly coplanar with the C—OH moiety and the O=C—C—O(H) torsion angle is 5.5 (2)°. An intra­molecular O—H⋯O hydrogen bond occurs. In the crystal, O—H⋯O hydrogen bonds connect the mol­ecules into supra­molecular chains along the *a-*axis direction.

## Related literature

For the crystal structure of 2-hy­droxy-2-(trifluoro­meth­yl)proprionic acid, see: Soloshonok *et al.* (2007 ▶). For background to chelate ligands, see: Gade (1998 ▶). For graph-set analysis of hydrogen bonds, see: Etter *et al.* (1990 ▶); Bernstein *et al.* (1995 ▶).
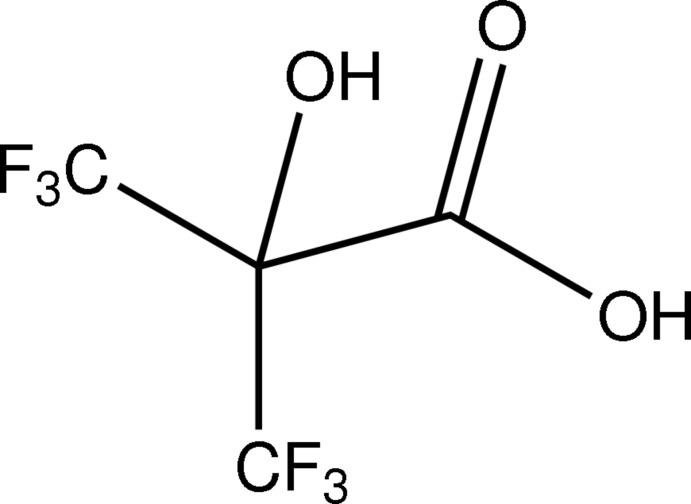


## Experimental

### 

#### Crystal data

C_4_H_2_F_6_O_3_
*M* *_r_* = 212.06
Orthorhombic, 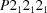

*a* = 5.9949 (2) Å
*b* = 6.4007 (2) Å
*c* = 18.5642 (6) Å
*V* = 712.34 (4) Å^3^
*Z* = 4
Mo *K*α radiation
μ = 0.26 mm^−1^
*T* = 200 K
0.53 × 0.53 × 0.34 mm

#### Data collection

Bruker APEXII CCD diffractometer
Absorption correction: multi-scan (*SADABS*; Bruker, 2008 ▶) *T*_min_ = 0.908, *T*_max_ = 1.000
3824 measured reflections
1052 independent reflections
1024 reflections with *I* > 2σ(*I*)
*R*_int_ = 0.010

#### Refinement

*R*[*F* ^2^ > 2σ(*F* ^2^)] = 0.029
*wR*(*F* ^2^) = 0.076
*S* = 1.05
1052 reflections
120 parameters
H-atom parameters constrained
Δρ_max_ = 0.31 e Å^−3^
Δρ_min_ = −0.18 e Å^−3^

### 

Data collection: *APEX2* (Bruker, 2010 ▶); cell refinement: *SAINT* (Bruker, 2010 ▶); data reduction: *SAINT*; program(s) used to solve structure: *SHELXS97* (Sheldrick, 2008 ▶); program(s) used to refine structure: *SHELXL97* (Sheldrick, 2008 ▶); molecular graphics: *ORTEP-3 for Windows* (Farrugia, 2012 ▶) and *Mercury* (Macrae *et al.*, 2008 ▶); software used to prepare material for publication: *SHELXL97* and *PLATON* (Spek, 2009 ▶).

## Supplementary Material

Crystal structure: contains datablock(s) I, global. DOI: 10.1107/S1600536813003103/tk5193sup1.cif

Supplementary material file. DOI: 10.1107/S1600536813003103/tk5193Isup2.cdx

Structure factors: contains datablock(s) I. DOI: 10.1107/S1600536813003103/tk5193Isup3.hkl

Supplementary material file. DOI: 10.1107/S1600536813003103/tk5193Isup4.cml

Additional supplementary materials:  crystallographic information; 3D view; checkCIF report

## Figures and Tables

**Table 1:** Hydrogen-bond geometry (Å, °)

*D*—H⋯*A*	*D*—H	H⋯*A*	*D*⋯*A*	*D*—H⋯*A*
O3—H3⋯O2	0.84	2.13	2.6274 (17)	118
O1—H1⋯O3^i^	0.84	1.91	2.7170 (17)	160
O3—H3⋯O2^ii^	0.84	2.06	2.7186 (17)	135

Symmetry codes: (i) 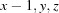; (ii) 
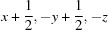
.

**Fig. 1.**
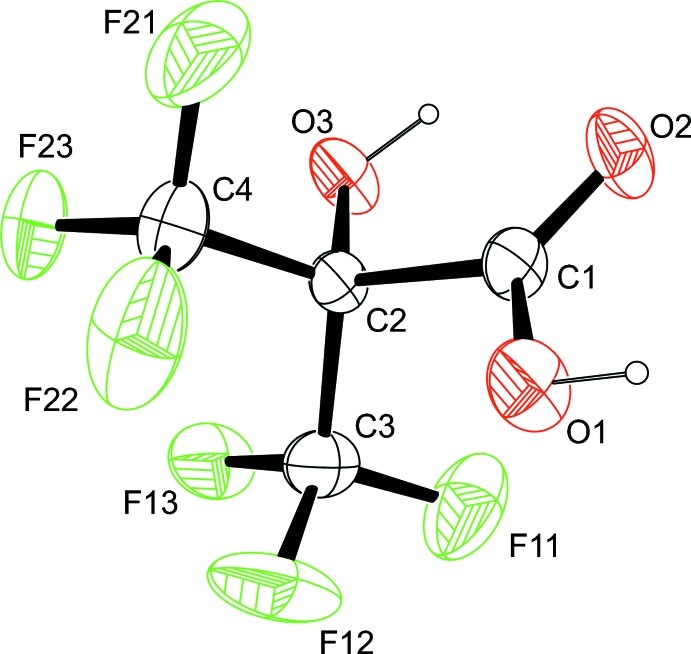
The molecular structure of the title compound, with atom labels and anisotropic displacement ellipsoids (drawn at 50% probability level).

**Fig. 2.**
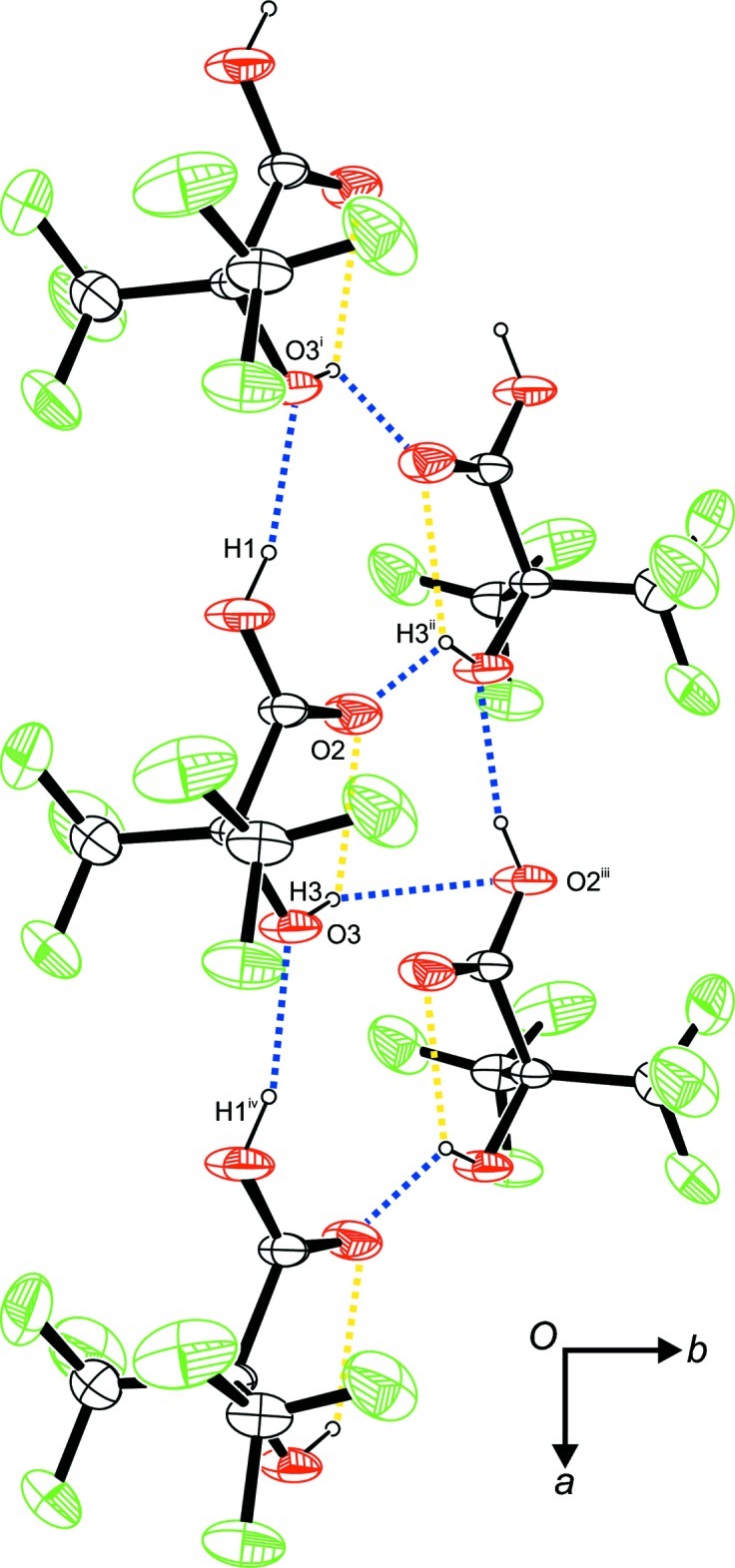
Intermolecular contacts, viewed along [0 0 - 1]. Blue dashed lines indicate intermolecular hydrogen bonds, yellow dashed lines indicate intramolecular hydrogen bonds. Symmetry operators: ix - 1, y, z; iix - 1/2, -y + 1/2, -z; iiix + 1/2, -y + 1/2, -z; ivx + 1x, y, z.

**Fig. 3.**
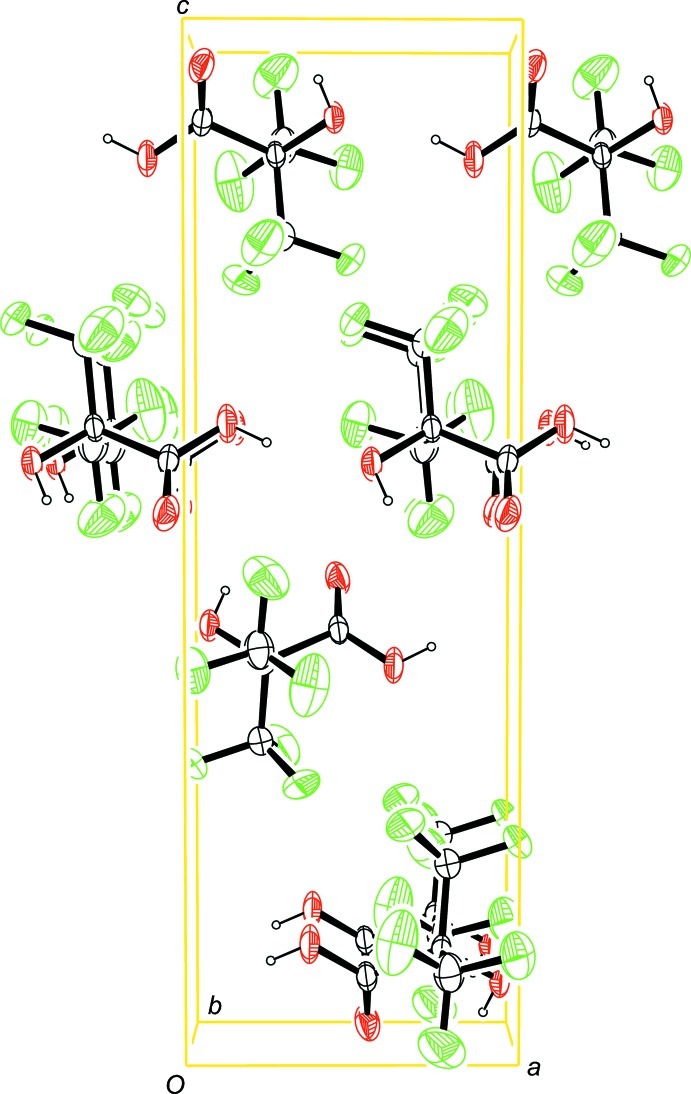
Molecular packing of the title compound, viewed along [0 1 0] (anisotropic displacement ellipsoids drawn at 50% probability level).

## supplementary crystallographic information

### Comment

Chelate ligands have found widespread use in coordination chemistry due to the increased stability of coordination compounds they can form in comparison to monodentate ligands (Gade, 1998). Hydroxycarboxylic acids are particularily interesting in this aspect as they offer two hydroxyl groups of markedly different acidity as potential bonding partners. Upon varying the substitution pattern on the hydrocarbon backbone, the acidity of the respective hydroxyl groups can be finetuned over a wide range and they may, thus, serve as probes for establishing the rules in which pK_a_ range coordination to various central atoms can be observed. To allow for comparisons of metrical parameters of the carboxylic-acid-derived ligand in envisioned coordination compounds, the crystal and molecular structure of the free ligand was determined. The crystal structure of a related compound, 2-hydroxy-2-(trifluoromethyl)proprionic acid, is apparent in the literature (Soloshonok *et al.*, 2007).

The carboxyl group is nearly in plane with the C–OH moiety. The respective O═C–C–O(H) torsion angle was found to be only 5.5 (2)°. This common plane also acts as internal mirror plane for the compound which shows approximately *C*_s_ symmetry (Fig. 1).

In the crystal, intermolecular hydrogen bonds can be observed. These are established between the carboxylic acid group as donor and the hydroxyl group as acceptor. The latter group itself, at the same time, acts as donor towards the double-bonded oxygen atom of the carboxyl group. The small dihedral angle among the O═C–C–O(H) moiety may be indicative of an intramolecular hydrogen bond as well, denoting the alcoholic hydroxyl group to give rise to a bifurcated hydrogen bond. Metrical parameters as well as information about the symmetry of these hydrogen bonds is summarized in Table 1. In terms of graph-set analysis (Etter *et al.*, 1990; Bernstein *et al.*, 1995), the descriptor for the hydrogen bonds is *S*(5)*C*^1^_1_(5)*C*^1^_1_(5) on the unary level (Fig. 2).

The packing of the title compound in the crystal structure is shown in Fig. 3.

### Experimental

The compound was obtained from Alfa Aesar. Crystals suitable for the diffraction study were taken directly from the provided product.

### Refinement

The H atoms of the hydroxyl groups were allowed to rotate with a fixed angle around the C—O bond to best fit the experimental electron density (HFIX 147 in the *SHELX* program suite (Sheldrick, 2008), with O—H = 0.84 Å, and with *U*(H) = 1.5*U*_eq_(O).

### Crystal data

**Table:**

C_4_H_2_F_6_O_3_	*F*(000) = 416
*M**_r_* = 212.06	*D*_x_ = 1.977 Mg m^−^^3^
Orthorhombic, *P*2_1_2_1_2_1_	Mo *K*α radiation, λ = 0.71073 Å
Hall symbol: P 2ac 2ab	Cell parameters from 2971 reflections
*a* = 5.9949 (2) Å	θ = 4.4–28.3°
*b* = 6.4007 (2) Å	µ = 0.26 mm^−^^1^
*c* = 18.5642 (6) Å	*T* = 200 K
*V* = 712.34 (4) Å^3^	Block, colourless
*Z* = 4	0.53 × 0.53 × 0.34 mm

### Data collection

**Table:**

Bruker APEXII CCD diffractometer	1052 independent reflections
Radiation source: fine-focus sealed tube	1024 reflections with *I* > 2σ(*I*)
Graphite monochromator	*R*_int_ = 0.010
φ and ω scans	θ_max_ = 28.3°, θ_min_ = 3.4°
Absorption correction: multi-scan (*SADABS*; Bruker, 2008)	*h* = −5→8
*T*_min_ = 0.908, *T*_max_ = 1.000	*k* = −6→8
3824 measured reflections	*l* = −22→24

### Refinement

**Table:**

Refinement on *F*^2^	Primary atom site location: structure-invariant direct methods
Least-squares matrix: full	Secondary atom site location: difference Fourier map
*R*[*F*^2^ > 2σ(*F*^2^)] = 0.029	Hydrogen site location: inferred from neighbouring sites
*wR*(*F*^2^) = 0.076	H-atom parameters constrained
*S* = 1.05	*w* = 1/[σ^2^(*F*_o_^2^) + (0.0385*P*)^2^ + 0.2376*P*] where *P* = (*F*_o_^2^ + 2*F*_c_^2^)/3
1052 reflections	(Δ/σ)_max_ < 0.001
120 parameters	Δρ_max_ = 0.31 e Å^−^^3^
0 restraints	Δρ_min_ = −0.18 e Å^−^^3^

### Special details

**Table:**

Refinement. Due to the absence of a strong anomalous scatterer, the Flack parameter is meaningless. Thus, Friedel opposites (636 pairs) have been merged and the item was removed from the CIF.

### Fractional atomic coordinates and isotropic or equivalent isotropic displacement parameters (Å^2^)

**Table:**

	*x*	*y*	*z*	*U*_iso_*/*U*_eq_	
F11	0.7286 (3)	0.2280 (3)	0.20540 (9)	0.0635 (5)	
F12	0.6570 (2)	−0.0851 (3)	0.23660 (7)	0.0670 (5)	
F13	0.99402 (19)	0.0068 (3)	0.21779 (6)	0.0464 (3)	
F21	0.7614 (3)	−0.2907 (3)	0.02996 (9)	0.0702 (5)	
F22	0.6292 (3)	−0.3692 (2)	0.13324 (12)	0.0747 (6)	
F23	0.9814 (2)	−0.3310 (2)	0.11960 (9)	0.0527 (4)	
O1	0.37188 (19)	−0.0103 (3)	0.11897 (8)	0.0403 (4)	
H1	0.2541	0.0380	0.1010	0.060*	
O2	0.5456 (2)	0.1808 (2)	0.03438 (8)	0.0372 (3)	
O3	0.94390 (19)	0.0739 (2)	0.08055 (7)	0.0338 (3)	
H3	0.8967	0.1464	0.0461	0.051*	
C1	0.5428 (3)	0.0629 (3)	0.08432 (9)	0.0260 (3)	
C2	0.7654 (3)	−0.0219 (3)	0.11475 (8)	0.0223 (3)	
C3	0.7855 (3)	0.0308 (4)	0.19560 (10)	0.0340 (4)	
C4	0.7833 (4)	−0.2590 (3)	0.10055 (12)	0.0382 (4)	

### Atomic displacement parameters (Å^2^)

**Table:**

	*U*^11^	*U*^22^	*U*^33^	*U*^12^	*U*^13^	*U*^23^
F11	0.0602 (10)	0.0626 (9)	0.0677 (9)	0.0226 (9)	−0.0209 (8)	−0.0336 (7)
F12	0.0487 (8)	0.1198 (14)	0.0326 (6)	−0.0194 (10)	0.0099 (6)	0.0174 (8)
F13	0.0276 (5)	0.0770 (9)	0.0347 (5)	0.0062 (7)	−0.0112 (4)	0.0026 (7)
F21	0.0736 (10)	0.0738 (10)	0.0632 (8)	0.0260 (10)	−0.0204 (8)	−0.0368 (8)
F22	0.0546 (9)	0.0326 (6)	0.1368 (17)	−0.0094 (7)	0.0194 (11)	0.0087 (9)
F23	0.0412 (7)	0.0433 (7)	0.0735 (9)	0.0204 (6)	−0.0056 (7)	0.0015 (7)
O1	0.0129 (5)	0.0608 (9)	0.0473 (7)	0.0005 (6)	0.0007 (5)	0.0171 (8)
O2	0.0213 (5)	0.0479 (8)	0.0424 (7)	0.0020 (6)	−0.0061 (5)	0.0185 (6)
O3	0.0133 (5)	0.0512 (8)	0.0368 (6)	−0.0008 (6)	0.0012 (5)	0.0206 (6)
C1	0.0142 (7)	0.0332 (8)	0.0305 (7)	0.0003 (7)	−0.0022 (6)	0.0021 (7)
C2	0.0127 (6)	0.0287 (7)	0.0254 (6)	0.0005 (6)	0.0014 (5)	0.0054 (6)
C3	0.0223 (8)	0.0501 (11)	0.0295 (8)	0.0020 (9)	−0.0016 (6)	−0.0007 (8)
C4	0.0301 (9)	0.0329 (9)	0.0517 (11)	0.0049 (8)	−0.0019 (9)	−0.0029 (9)

### Geometric parameters (Å, º)

**Table:**

F11—C3	1.320 (3)	O1—H1	0.8400
F12—C3	1.313 (3)	O2—C1	1.196 (2)
F13—C3	1.325 (2)	O3—C2	1.3870 (19)
F21—C4	1.332 (3)	O3—H3	0.8400
F22—C4	1.312 (3)	C1—C2	1.547 (2)
F23—C4	1.322 (2)	C2—C3	1.543 (2)
O1—C1	1.297 (2)	C2—C4	1.544 (3)
			
C1—O1—H1	109.5	F12—C3—F13	107.93 (17)
C2—O3—H3	109.5	F11—C3—F13	108.19 (19)
O2—C1—O1	128.59 (16)	F12—C3—C2	113.24 (17)
O2—C1—C2	119.48 (15)	F11—C3—C2	108.83 (17)
O1—C1—C2	111.93 (13)	F13—C3—C2	110.55 (15)
O3—C2—C3	106.75 (14)	F22—C4—F23	108.76 (18)
O3—C2—C4	107.61 (15)	F22—C4—F21	107.7 (2)
C3—C2—C4	112.06 (15)	F23—C4—F21	107.36 (18)
O3—C2—C1	110.08 (12)	F22—C4—C2	113.62 (17)
C3—C2—C1	110.23 (14)	F23—C4—C2	111.08 (17)
C4—C2—C1	110.02 (15)	F21—C4—C2	108.10 (17)
F12—C3—F11	107.96 (19)		
			
O2—C1—C2—O3	5.5 (2)	O3—C2—C3—F13	−44.9 (2)
O1—C1—C2—O3	−174.76 (17)	C4—C2—C3—F13	72.7 (2)
O2—C1—C2—C3	123.00 (19)	C1—C2—C3—F13	−164.42 (16)
O1—C1—C2—C3	−57.3 (2)	O3—C2—C4—F22	176.80 (17)
O2—C1—C2—C4	−112.92 (19)	C3—C2—C4—F22	59.8 (2)
O1—C1—C2—C4	66.81 (19)	C1—C2—C4—F22	−63.3 (2)
O3—C2—C3—F12	−166.13 (17)	O3—C2—C4—F23	53.8 (2)
C4—C2—C3—F12	−48.6 (2)	C3—C2—C4—F23	−63.2 (2)
C1—C2—C3—F12	74.3 (2)	C1—C2—C4—F23	173.74 (15)
O3—C2—C3—F11	73.80 (18)	O3—C2—C4—F21	−63.7 (2)
C4—C2—C3—F11	−168.63 (17)	C3—C2—C4—F21	179.21 (16)
C1—C2—C3—F11	−45.7 (2)	C1—C2—C4—F21	56.2 (2)

### Hydrogen-bond geometry (Å, º)

**Table:**

*D*—H···*A*	*D*—H	H···*A*	*D*···*A*	*D*—H···*A*
O3—H3···O2	0.84	2.13	2.6274 (17)	118
O1—H1···O3^i^	0.84	1.91	2.7170 (17)	160
O3—H3···O2^ii^	0.84	2.06	2.7186 (17)	135

Symmetry codes: (i) *x*−1, *y*, *z*; (ii) *x*+1/2, −*y*+1/2, −*z*.
